# 

**DOI:** 10.1192/bjb.2021.13

**Published:** 2021-08

**Authors:** Oluwadamilola Ajayi

**Affiliations:** Member of the Faculty of Psychiatry of the West Africa College of Physicians and an Associate Fellow of the National Post-Graduate Medical College of Nigeria. Currently, he is participating in the Royal College of Psychiatrists’ Medical Training Initiative, working at St Charles Centre for Health Centre and Well Being, London, UK. Email: ajayidami@gmail.com

Malawi, a south-eastern African country of approximately 20 million people, has only three consultant psychiatrists. As grim as this ratio is, it is not unique to Malawi; rather, it is a tendency within Africa that begs interventions.

*The Malawi Quick Guide to Mental Health*, by the Scotland–Malawi Mental Health Education project, is one such intervention. With Donncha S. Mullin and Robert C. Stewart as lead editors, this is a seven-part practical resource material adapted mostly from World Health Organization's *mhGAP Intervention Guide Version 2.0* (2016) and the Royal College of Psychiatrists’ *Where There is No Psychiatrist* (2018).

The target reader is ‘the busy primary care provider working at first- and second-level healthcare facilities in Malawi’ and the 90-page guide is adapted to the practical realities of working in mental health, paying attention to the country's official national language (Chichewa), social mores, current mental health legislation and local support institutions.

Written in accessible language and formatted in bullet-point presentation style, the first three parts provide guidance about mental health and the ongoing COVID-19 pandemic, mental health emergencies and the principles of assessment and management of mental illnesses. The other sections offer guidance about specific psychiatric disorders, including epilepsy, which is identified within the neurologist's remit but may present to mental health services.

A chapter is dedicated to special populations (pregnant women, older adults, children and adolescents) and a final part contains information leaflets on specific mental disorders written in English and Chichewa, adapted from existing leaflets of the Royal College of Psychiatrists, Mind and National Centre for Mental Health.

This guide fulfils its aim in providing support for the non-specialist mental health worker in landlocked Malawi and neighbouring Anglophone countries, by virtue of its being written in English. Always signposting to other resource materials for additional information, this guide is a primer for the curious non-specialist health worker.

The curious specialist reader will be furnished with aspects of the Malawian world-view about mental illness and the country's mental health legislation, the Malawi Mental Health Treatment Act 1948, a functional colonial relic, which is also not unique to Malawi. *The Malawi Quick Guide to Mental Health* is a pragmatic intervention addressing the dearth of mental health specialists in Malawi but this resource material will also serve other Anglophone low- and middle-income countries in similar predicaments.

